# Comparison of the effect of low level laser therapy with alvogyl on the management of alveolar osteitis

**DOI:** 10.4317/medoral.20375

**Published:** 2015-02-07

**Authors:** Majid Eshghpour, Farzaneh Ahrari, Navab-Teymour Najjarkar, Mohammad-Amin Khajavi

**Affiliations:** 1 DDS, MS. Dental Research Center, School of Dentistry, Mashhad University of Medical Sciences, Mashhad, Iran; 2 DDS. School of Dentistry, Mashhad University of Medical Sciences, Mashhad, Iran

## Abstract

**Background:**

This study investigated the efficacy of low level laser therapy (LLLT) for managing alveolar osteitis (AO).

**Material and Methods:**

Sixty patients with alveolar osteitis of mandibular third molars were randomly divided into three groups. In group 1, socket irrigation was followed by alvogyl placement, and the treatment was repeated 48 hours later. In group 2, socket was irradiated with a low power red laser for 3 consecutive days (200 mW, 30 seconds on each of the buccal and lingual surfaces and 30 seconds at the middle of the socket, 6 J per area). The subjects in group 3 underwent treatment with a low power infrared laser with the same parameters as group 2. A visual analogue scale (VAS) was used to record the degree of pain at the morning (T0, before intervention) and at 6 (T1) and 12 (T2) hours later for 3 days.

**Results:**

Pain was significantly lower in the alvogyl group than the other groups at T1 and T2 points on day 1 and at T0 and T1 points on day 2 (*p*<0.05). At T2 point on day 2 and on day 3, VAS became significantly lower in the red laser group compared to the other groups (*p*<0.05). The infrared laser was not more efficacious than the other groups at any of the treatment intervals, but it reduced VAS to an acceptable level.

**Conclusions:**

LLLT displayed good results in this study for treatment of alveolar osteitis and should be further investigated as an alternative to alvogyl for AO management.

**Key words:** Low level laser, low power laser, therapy, alvogyl, dry socket, alveolar osteitis, mandibular third molar.

## Introduction

Alveolar osteitis (AO) or dry socket is the most common complication of dental extraction. It occurs with the incidence of 1-4% after routine dental extractions (1,2), although its incidence may reach 30% following surgical removal of impacted mandibular third molars (2,3). The experience of an unbearable pain that is not easily alleviated with analgesics and the presence of an empty socket due to the degradation of blood clot are the characteristic features of this condition. Furthermore, marked halitosis, foul taste and regional lymphadenitis are frequently observed in patients affected with AO. The condition typically initiates 1-3 days after extraction and remains for 7 to 10 days, during which the socket is being covered with new granulation tissue (2). The most commonly reported risk factors for dry socket formation include age (2), gender (2), site of extraction (2), difficult extraction (4), smoking (5-8), pericoronitis (9), use of oral contraceptives (8-10) and menstrual cycle (8,11). Despite all the measures that have been suggested to prevent AO, this condition continues to occur in every dental practice and make the patients unsatisfied with the treatment results. Therefore, finding an ideal approach for managing dry socket is considered a subject of interest.

Because of the severe pain, the treatment strategies for AO are mainly palliative and are based on attenuating patient’s pain/discomfort over the period that healing spontaneously occurs. However, accelerating the healing process should also be considered as the secondary aim of the treatment. A widely used dressing for management of AO is alvogyl which contains eugenol (analgesic and anti-inflammatory), iodoform (antimicrobial), and butamen (anesthetic) (12). Other therapeutic agents such as zinc oxide eugenol paste (13), SaliCept patch (12) or plasma rich in growth factor (PRGF) with gelatin sponge (13) have also been used successfully for AO management.

With introducing low power lasers, their applications have been remarkably increased in different aspects of dentistry including oral surgery procedures. Low level laser therapy (LLLT) has a proven efficacy in accelerating the wound healing process (14,15), reducing pain (16,17) and shortening the duration of the inflammatory phases (16). These various biochemical effects of LLLT suggest that it may be a suitable treatment alternative to the conventional methods for managing dry socket, which can act not only by reducing pain but also through acceleration of the healing process.

There are only few studies regarding the effectiveness of LLLT in the treatment of dry socket and comparison of low power red and infrared lasers for this purpose has not been performed according to the authors’ knowledge. Therefore, this study was conducted to compare the effects of low level red and infrared lasers and alvogyl in managing patients affected with dry socket.

## Material and Methods

The sample consisted of sixty patients affected with alveolar osteitis of mandibular third molars, who referred to the Department of Oral and Maxillofacial Surgery of Mashhad Dental School, Mashhad University of Medical Sciences, Mashhad, Iran. The patients were 34 females and 26 males with mean age of 35.3 years (age range 23 to 65 years). The inclusion criteria consisted of subjects with at least 18 years old age in whom the definite diagnosis of dry socket in a mandibular third molar was made by an oral and maxillofacial surgeon according to the following criteria: the presence of severe pain in and around the socket initiated 1 to 3 days post operatively, and the observation of an alveolar socket devoid of blood clot with exposed bone. The study design excluded patients who had a background of radiotherapy or were affected with systemic diseases that could interact with the treatment process such as bone pathologic conditions, hematologic diseases, diabetes mellitus and etc. Furthermore, subjects who were smokers, affected with psychiatric disorders or had conditions that laser therapy could be contraindicated such as pregnancy were excluded from the sample. The protocol of the study was reviewed and approved by the Ethics Committee of Mashhad University of Medical Sciences, and each participant signed and informed consent document after being aware of the treatment process.

This was a prospective randomized clinical trial. The participants were divided into three groups of 20 each according to a random number table, and underwent the following treatments:

- Group 1 (Alvogyl): In this group, after local anesthesia with 2% lidocaine combined with 1:100,000 epinephrine, the extraction socket was thoroughly irrigated with 0.9 % sterile saline solution to remove debris and bacteria from the denuded bone. Afterwards, a sufficient amount of alvogyl (Septodont, France) was inserted within the socket. Saline irrigation was repeated 48 hours later and alvogyl was refreshed.

- Group 2 (Low power red laser; LPRL): The patients in this group received treatment from a low power indium-gallium-aluminum-phosphide (InGaAlP) diode laser (Thor DD2 control unit, Thor, London, UK) for 3 consecutive days. The outcome of our pilot study (the data have not been presented) revealed that laser therapy on every other day resulted in severe pain experience between irradiations, so LLLT was performed on consecutive days. The apparatus emitted a wavelength of 660 nm and operated at the power of 200 mW and continuous-wave mode. The irradiation was accomplished on three areas (buccal and lingual surfaces of the socket and at the middle of the socket) for 30 seconds each, delivering 6 J of energy per area. The energy density at the surface of the probe was calculated to be 85.7 J/cm2 (surface area of the probe, 0.07 cm2). However, the probe was held at an approximate distance of 1 cm and the beam was divergent, so the energy density at the target area would be lower than that calculated at the surface of the probe. Both patient and therapist wore safety goggles during treatment.

- Group 3 (Low power infrared laser; LPIL): The device used in this group was a low level gallium-aluminum-arsenide (GaAlAs) diode laser (Thor), emitting a wavelength of 810 nm. Similar to group 2, the laser was applied at the power of 200 mW and continuous-wave mode and irradiation was performed for 30 seconds on each target area (buccal and lingual surfaces of the socket and at the middle of the socket). The energy of 6 J was delivered per area, with energy density of 21.4 J/cm2 considering the spot size of 0.28 cm2 at the surface of the probe. However, the laser hand piece was approximately 1 cm far from the target area and so the actual energy density would be lower than that calculated at the probe surface. The patients attended therapy for 3 consecutive days and eye protection was taken with safety goggles.

-Outcome assessment

The patients were followed-up for 3 days after diagnosis and initiating treatment measures. Each patient was requested to record the pain level experienced at the morning (T0, before intervention) and at 6 (T1) and 12 (T2) hours later for 3 days. A visual analogue scale (VAS) was used to quantify pain, consisting of a 10-cm horizontal scale, with 0 (the left side) indicating the absence of pain and 10 (the right side) representing the most severe pain possible. Additional follow-ups were set for some patients if required.

-Statistical analysis

The Kolmogorov-Smirnov test indicated that the pain data relating to the first day of the experiment were normally distributed (*p*>0.05), while those of days 2 and 3 were not (*p*<0.05). Therefore, a repeated measures analysis was run to detect any significant differences in pain intensity between the study groups and between the different evaluation times in each group on day 1. For days 2 and 3, the Kruskal-Wallis test, Mann-Whitney U test (with Bonferroni correction), Friedman test and Wilcoxon signed-rank test were used for inter- and intra-group comparisons of pain level. The statistical calculation was performed by SPSS (Statistical Package for Social Sciences, Version 16.0, SPSS Inc, Chicago, IL) software, and the significance level was determined at *p*<0.05.

## Results

- Comparison of pain intensity between the different evaluation times in each group

-Day 1

**Figure 1:**
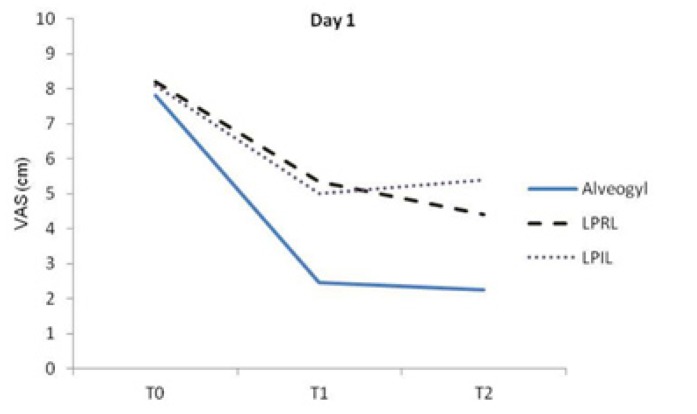
A line chart indicating VAS values in the study groups at different evaluation times on day 1. Significant differences were found between T0-T1 and T0-T2 in the alvogyl and LPIL groups and between T0-T1, T0-T2 and T1-T2 in the LPRL group (*p*<0.05).

The repeated measures analysis revealed significant differences in VAS scores between the different evaluation times in each group (*p*<0.001). Figure 1 represents variations of pain intensity in the study groups before intervention (T0) and at 6 (T1) and 12 hours (T2) later on day 1. The alvogyl group showed a remarkable decrease in pain after 6 hours of intervention (*p*<0.05) with no significant alteration between 6 and 12 hours later. Both laser groups also showed significant decreases in VAS scores after 6 hours of intervention (*p*<0.05). Between T1 and T2 time points, VAS significantly decreased in the LPRL group (*p*<0.05), but in the LPIL group, a negligible increase in pain occurred (Fig. 1).

-Day 2 

**Figure 2:**
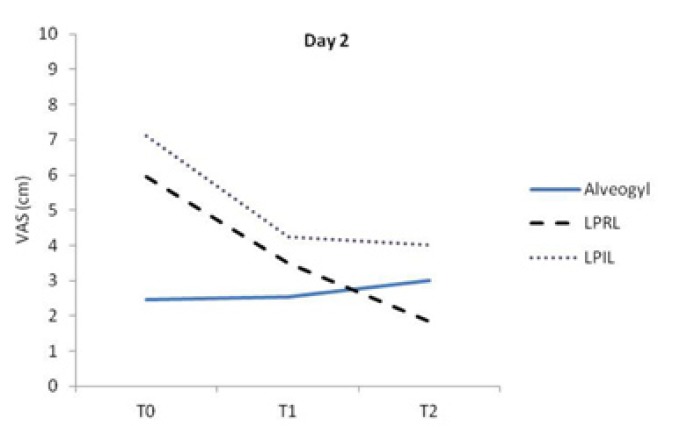
A line chart indicating VAS values in the study groups at different evaluation times on day 2. Significant differences were found between T0-T2 and T1-T2 in the alvogyl group (pain worsening), between T0-T1, T0-T2 and T1-T2 in the LPRL group and between T0-T1 and T0-T2 in the LPIL group (*p*<0.05).

The Friedman test revealed significant differences in pain between the different treatment intervals in each group (*p*<0.05). Figure 2 demonstrates variations of VAS scores in the study groups at T0 and at 6 (T1) and 12 hours (T2) later on day 2. Pain was slightly but significantly worsened in the alvogyl group between T0-T2 and T1-T2 time points (*p*<0.05). On the other hand, there was a significant reduction in painful symptoms after 6 hours of intervention in both laser groups (*p*<0.05), which continued until T2 time point in the LPRL group (Fig. 2).

-Day 3

**Figure 3:**
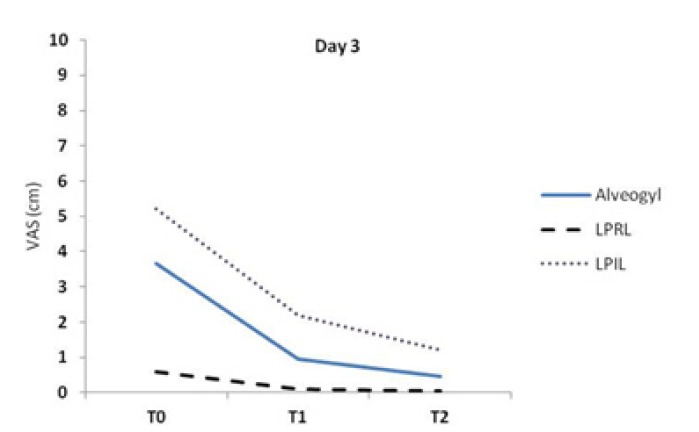
A line chart indicating VAS values in the study groups at different evaluation times on day 3. Significant differences were found between T0-T1, T0-T2 and T1-T2 in the alvogyl and LPIL groups and between T0-T1 and T0-T2 in the LPRL group (*p*<0.05).

The Friedman test indicated significant differences in pain between the different evaluation times in each group (*p*<0.05). The variations of VAS scores on day 3 are demonstrated in figure 3. All groups experienced significant decreases in painful symptoms between the treatment intervals (*p*<0.05) with the exception of LPRL group between T1-T2 time points (*p*>0.05). The LPRL group was the only one that completed the study period with a pain value near zero (Fig. 3).

- Comparison of pain intensity between the study groups at each treatment interval 

-Day 1

**Figure 4:**
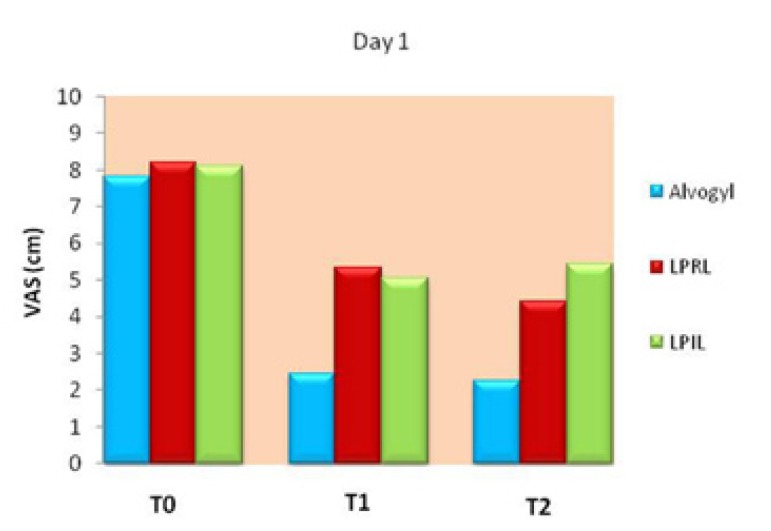
Comparison of pain intensity among the study groups on day 1. Significant differences were found between Alvogyl and both laser groups at T1, and between all the study groups at T2 (*p*<0.05).

Fig. 4 compares the VAS scores of the study groups at different time points on day 1. Analyzing the data with repeated measures analysis reveled a significant interaction between the two variables of time and group (*p*<0.001), so one way analysis of variance (ANOVA) was run to compare pain intensity among the study groups at each treatment interval. The results showed that the pretreatment pain (T0) was not significantly different among the groups (*p*=0.509), but at both 6 (T1) and 12 (T2) hours after intervention, significant between-group differences were found (*p*<0.001). Tukey pairwise comparison test revealed that at both T1 and T2, pain was significantly lower in the alvogyl group compared to the red and infrared laser groups (*p*<0.05). At T1, the two laser groups were not significantly different from each other, but at T2, pain was significantly lower in the red compared to the infrared laser group (*p*<0.05; Fig. 4).

-Day 2

**Figure 5:**
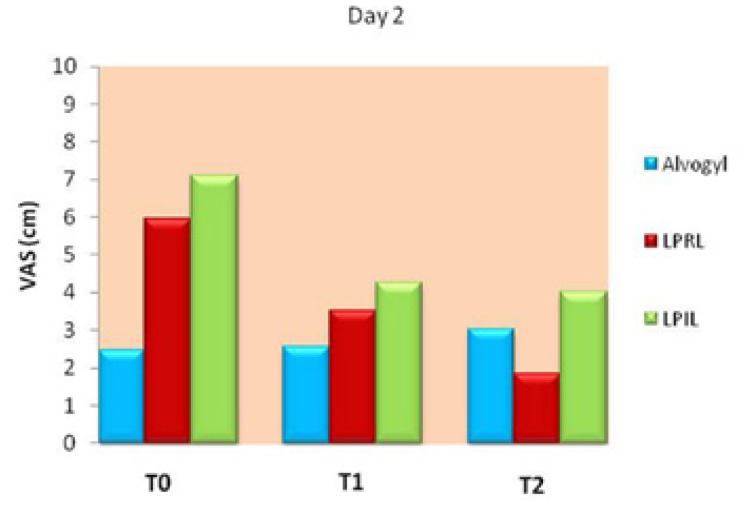
Comparison of pain intensity among the study groups on day 2. Significant differences were found between all the study groups at T0 and T2 time points, and between Alvogyl and both laser groups at T1 (*p*<0.01).

Figure 5 presents comparison of VAS scores among the study groups at different time points on day 2. Significant differences were found in VAS scores among the study groups at all treatment intervals on day 2 (*p*<0.001). Pair wise comparisons indicated that at T0, pain was significantly lower in the alvogyl group than the red laser group, which in turn showed significantly lower pain than the infrared laser group (*p*<0.01). At T1, the mean VAS was significantly lower in the alvogyl than both laser groups (*p*<0.01), which were not significantly different from each other. At T2, the VAS became significantly lower in the red laser group than the other groups, and it was significantly lower in the alvogyl group than the infrared laser group (*p*<0.01; Fig. 5).

-Day 3 

**Figure 6:**
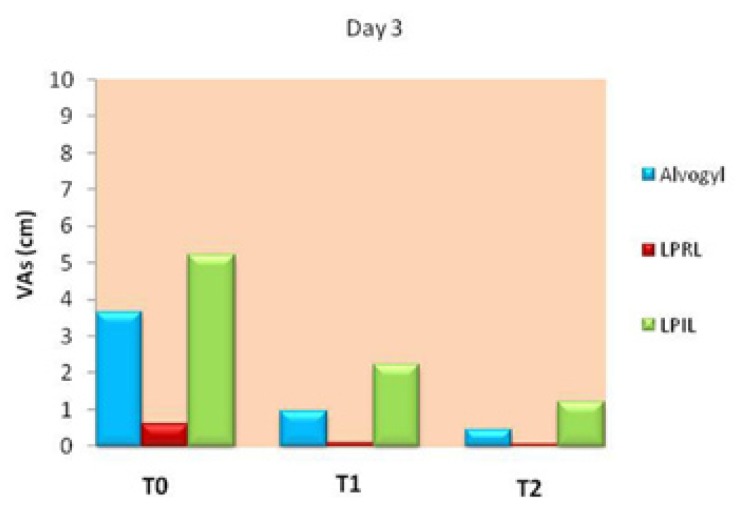
Comparison of pain intensity among the study groups on day 3. Significant differences were found between all the study groups at T0, T1 and T2 (*p*<0.01).

Comparison of VAS scores among the study groups on day 3 is presented in figure 6. There were significant between-group differences in VAS scores at all treatment intervals on day 3 (*p*<0.001). The Mann Whitney U test indicated that at T0, T1 and T2, pain intensity in the red laser group was significantly lower than both the alvogyl and infrared laser groups, and in the alvogyl group, it was significantly lower than the infrared laser group (*p*<0.01; Fig. 6).

## Discussion

The present study compared the clinical outcomes of low level red and infrared lasers with that of alvogyl for the treatment of dry socket. Alvogyl was employed following socket irrigation on the first and third days of the experiment and caused a rapid alleviation of pain. On the first day, the mean VAS decreased from 7.81 to 2.45 degree (69 % improvement) after 6 hours, while between 6 and 12 hours of intervention, it decreased from 2.45 to 2.25 (8 % improvement). On the second day, the degree of pain increased 4% and 18% after 6 hours and between 6 and 12 hours of initial examination, respectively. Although pain worsening was small in the alvogyl group on day 2, it reached statistical significance between T1-T2 and T0-T2 time points. On the third day, the mean VAS significantly decreased after refreshing the dressing, so that pain improved 74% within the first 6 hours and about 53% between 6 and 12 hours of intervention. The effectiveness of dressings containing eugenol in management of patients with postoperative pain has been reported in previous studies (1,18). The lower efficacy of alvogyl in decreasing pain between 6 and 12 hours of intervention and also its lack of effect on day 2 indicate that although alvogyl lessens patients’ pain immediately, but its effect is not maintained over time.

In the second group of this study, the application of 660 nm laser caused a significant reduction in pain over the course of the experiment. Although the improvement was more evident in the first 6 hours after irradiation, but there was also a remarkable improvement between 6 and 12 hours later, implying that the biomodulative effects of LPRL remain for several hours after its application. On day 1, VAS decreased from 8.21 to 5.35 degree (35% improvement) after 6 hours of intervention, and from 5.35 to 4.42 (17% improvement) between 6 and 12 hours later. On day 2, VAS improved 41% and 47% after 6 hours and between 6 and 12 hours of laser therapy, respectively. The VAS scores on day 3 were near zero and were the lowest among the study groups.

LLLT was performed with an 810 nm infrared laser in the third group of this experiment. On the first day, pain decreased 38% (VAS reduction from 8.11 to 5.05 degree) after the first 6 hours of laser application, while between 6 and 12 hours later, not only pain reduction stopped but also there was 7% worsening in the pain experienced by the patients. The VAS improved 40% following 6 hours, and 5% between 6 and 12 hours of irradiation on day 2. The corresponding values were 57% and 45% on day 3. The total amount of reduction in VAS scores on days 1, 2 and 3 was 2.7, 3.1 and 4 degree, respectively, which means that the efficacy of the 810 nm diode laser increased over time.

Comparison of pain intensity among the study groups revealed that on the first day, pain was significantly lower in the alvogyl group than the other groups at both 6 and 12 hours after intervention. This difference also existed at T0 and T1 time points on day 2, when significantly lower pain was experienced by the patients in the alvogyl group compared to the other groups. This can be attributed to the presence of the dressing that could fill the empty socket and in this way prevent from stimulation of denuded bone, which is one of the main factors producing pain. Furthermore, the dressing contains analgesic and local anesthetic factors that can act immediately and reduce patients’ pain. However, the presence of a dressing within the extraction socket could also be associated with delayed healing (19).

The order of pain intensity changed after 12 hours of intervention on day 2, so that the mean VAS became significantly lower in the red laser group than the other groups. The significantly lower pain intensity in the LPRL group continued at all treatment intervals on day 3 compared to the LPIL and alvogyl groups. These results indicate that the alveogyl has a more immediate effect than LLLT on reducing pain of the patients affected with dry socket, but after 12 hours of intervention on day 2 and at all treatment intervals on day 3, the 660 nm laser became more efficacious than the alvogyl for pain reduction. This may be related to the fact that LLLT is not only capable to reduce pain and inflammation, but also can increase the rate and quality of wound healing (14,15). The effect on wound healing can be achieved by several mechanisms such as stimulating the proliferation of fibroblasts (20), enhanced epithelization (21), improved organization and maturation of collagen fibers (14,22) and increased matrix synthesis and angiogenesis (14).

The infrared laser did not perform more effectively than the other groups at any of the treatment evaluations, but it caused a significant decrease in VAS scores during the 3-day treatment course, so that pain reached an acceptable value (VAS =1.2 degree) at the end of the experiment. It is possible that with repetitious use of this laser on the next days or with alteration in laser parameters, its efficacy would be improved, but further studies are warranted for verification.

It should be noted that in the control (alvogyl) group, the socket was gently irrigated with normal saline solution before placing alvogyl in order to remove debris and bacteria. In the LLLT groups, however, socket irrigation was not performed in order to eliminate the need for local anesthesia and to accelerate the treatment process. Furthermore, since no material was inserted in the socket, irrigation seemed to be not obligatory. Treatment of dry socket is a time-consuming process for both the surgeon and patient, which may require several appointments for refreshing the dressing. We aimed to reach an effective approach that could be accomplished by a laser therapist or a dental assistant without the need to interact with routine activities in the dental office. It is possible, however, that socket irrigation before laser therapy would improve the treatment outcomes, but further studies are required to elucidate this issue.

There are few studies regarding the effectiveness of LLLT in the treatment of AO. Jovanovic *et al*. (23) evaluated the effectiveness of low level laser therapy in pain relief and healing of extraction wounds with alveolar osteitis and reported that the LLLT group experienced significantly lower pain on days 5 to 8 after intervention compared to a control group in which a dressing of zinc oxide eugenol paste was applied. Kaya *et al*. (12) indicated that LLLT with an 808 nm diode laser at 7.64 J/cm2 performed quickly and superiorly compared to the alvogyl and SaliCept patch for the management of AO. The outcomes of this study are in contrast with those of Kaya *et al*. (12), because the LPIL group showed lower efficacy than the alvogyl group in pain reduction. This can be attributed to the use of different laser parameters in this study compared to that of Kaya *et al*. (12) or to the lack of socket irrigation in the laser groups of this study, while *Kaya et al*, performed curettage and irrigation in all of the study groups. If the current study was continued for a longer time, it was possible that the difference between the LPIL and the other groups would be insignificant. However, the immediate effect of the treatment modalities for dry socket should be considered more important than their delayed effect.

By comparing the effect of the two laser wavelengths used in this study, it was revealed that the red laser performed significantly better than the infrared laser at all treatment intervals, especially on days 2 and 3 of the experiment. This may be related to the high efficacy of the red laser wavelength for enhancing the wound healing process. The red laser is mainly absorbed in the first 7-8 mm of the tissue, while the penetration depth of the infrared laser reaches 2-3 cm. It seems that regarding dry socket, the surface absorption of laser energy is more important than its deep penetration into the alveolar bone. Another variable that could be important in the treatment results of low power lasers is the energy delivered to the target tissue and the energy density (dosage) (24). In both groups of LLLT, 6 J of energy was delivered to each area (18 J in total). The energy densities at the surface of the probes in this study were 85.6 J/cm2 for the red laser and 21.4 J/cm2 for the infrared laser groups. The dosage of the red laser was several times greater than the infrared laser and this can contribute to the different outcomes obtained with these two wavelengths. Bjordal *et al*. (16) believed that achieving analgesic and anti-inflammatory effects from infrared lasers requires at least 6 J/cm2 of energy density for small wounds and 10 J/cm2 for large wounds. However, in situations such as this study in which the laser is irradiated from above the socket and the target tissue has a relatively large distance from the probe, the exact calculation of energy density at the target area is not possible. The anatomic variations of the socket among patients, the invisible nature of the infrared laser, and the reduction in energy density because of the divergence of the beam contribute to this problem.

The limitation of this study was the short follow-up period. Further studies with longer follow-ups are warranted to find the optimum parameters of low power lasers and to evaluate the combined effects of red and infrared lasers in reducing pain and accelerating the healing process in patients affected with dry socket.

## Conclusions

Under the conditions used in this study.

1- The immediate effect of the alvogyl in reducing pain of the patients affected with dry socket was significantly greater than the low power red and infrared lasers, but after 12 hours of intervention on day 2 and at all treatment intervals on day 3, the 660 nm laser became more effective. Therefore, the low level red laser should be further investigated as an alternative to alvogyl in the management of AO.

2- Although pain significantly decreased in the 810 nm laser group over time, its performance was not better than either the alvogyl or the 660 nm laser groups at any of the treatment intervals.
